# Zero to minimal fluoroscopy for cardiac electronic device implantation: A systematic review and meta‐analysis

**DOI:** 10.1002/joa3.12949

**Published:** 2023-11-15

**Authors:** Kevin Luke, Clonia Milla, Joshua Kurnia Tandi, Rerdin Julario

**Affiliations:** ^1^ Faculty of Medicine Universitas Airlangga Surabaya Indonesia; ^2^ Faculty of Medicine Universitas Tarumanagara Jakarta Indonesia; ^3^ Department of Cardiology and Vascular Medicine Dr. Soetomo General Hospital‐Universitas Airlangga Surabaya Indonesia

**Keywords:** bradyarrhythmia, cardiac resynchronization therapy, fluoroscopy, heart failure, His bundle pacing

## Abstract

**Background:**

Fluoroscopy is conventionally performed for cardiac implantable electronic device (CIED) therapy and carries radiation drawback for both patients and medical workers. Recently, zero to minimal fluoroscopy (ZMF) approach is introduced to reduce radiation exposure of fluoroscopy. This study compares the feasibility and safety of ZMF approach to fluoroscopy for CIEDs therapy in adults.

**Method:**

A systematic literature search was conducted on PubMed, ScienceDirect, and Web of Science in March 2023. All observational or experimental studies comparing ZMF approach to fluoroscopy for adult CIEDs therapy were included. Reviews, case report/series, animal studies, and non‐English articles were excluded. The success rate, procedural time, fluoroscopy time, radiation dose, and complications rate were compared for each approach.

**Results:**

Seven articles for permanent and three articles for temporary CIEDs were included for analysis. The success rate of ZMF for permanent CIEDs was similar to fluoroscopy method (OR: 0.77, 95% CI: 0.33–4.15). The procedural time of ZMF was similar to fluoroscopy for both permanent and temporary CIEDs (standardized mean difference [SMD]: 0.10, 95% CI: −0.35 to 0.55 and SMD: −0.71, 95% CI: −1.87–0.44, respectively). However, ZMF approach markedly reduced the fluoroscopy time and radiation exposure for permanent CIEDs (SMD: −1.80, 95% CI: −2.49 to −1.12 and SMD: −1.26, 95% CI: −2.24 to −0.29). The complication rate was similar for permanent CIEDs (OR: 1.08, 95% CI: 0.41–2.84), yet lowered for temporary CIEDs (OR: 0.34, 95% CI: 0.20–0.59).

**Conclusion:**

ZMF had similar success rate, procedural time, and sum complication rate for permanent CIEDs implantation with a significant reduction of fluoroscopy time and radiation exposure.

## Graphical abstract

Current evidence suggests that Zero‐to‐minimal fluoroscopy is a promising alternative to fluoroscopy for CIED implantation in adults. It offers similar success rates, procedural times, and complication rates to fluoroscopy. Additionally, it provides the added benefit of significantly reducing fluoroscopy time and radiation exposure.

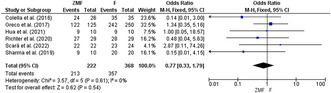

## 1 INTRODUCTION

Implantation of electronic cardiac devices (CIEDs) has been one of the most ideal solutions in both monitoring and controlling patients with cardiac arrhythmias and heart failure, showing benefits in reducing morbidity and mortality.^1^ As the field of cardiac interventional electrophysiology has been rapidly progressing, there have been a diverse amount of implantable cardiac devices for a variety of indications, from permanent pacemaker (PPM), implantable cardioverter‐defibrillator (ICD), to cardiac resynchronization therapy (CRT) devices.^2^ However, such procedure requires a guidance, making fluoroscopy the most commonly used imaging modality for CIEDs implantation in the past few decades.

Fluoroscopy is a radiology‐based modality used for both diagnostic and therapeutic purposes. This conventional method of imaging exposes both the patient and the operator during the CIEDs implantation procedure. The effective dose (ED) ranges around 4 millisievert (mSv) for PPM or ICD implant to 25 mSv for CRT implant, exposing 200–1100 times radiation than that of a single chest x‐ray, making a CIEDs operator more vulnerable than a diagnostic radiologist. To minimalize radiation exposure, there are a few approaches that can be done. The first and second methods were to reduce the time spent near the radiation source and to keep as much distance as possible, which were not very feasible for both the medical staff and the patient when fluoroscopy is performed.^3^ The only available method was by shielding. Nevertheless, even with the use of radioprotective gears, radiation exposure could only be reduced up to a thousand‐fold. A cumulative effective radiation dose of 100 mSv or higher was known to be correlated with a higher risk of cancer, which may be reached after a patient underwent four fluoroscopy procedures with two CT scans, equivalent to 30 years of work for an experienced cardiologist. Moreover, these populations were more prone to radiation‐associated injuries. In addition, not all patients in need of CIEDs implantation were allowed to undergo fluoroscopy, for example, pregnant women, patients with kidney diseases, and those with contrast allergies. Therefore, an alternative with comparable outcomes and minimal drawbacks to that of fluoroscopy is exigently in demand.^4^

Zero‐to‐minimal fluoroscopy (ZMF) has been deemed as an alternative to fluoroscopy, as a meta‐analysis demonstrated comparable success rates and complications but with less radiation exposure for catheter ablation procedures.^5^ However, to our knowledge, no meta‐analysis has been conducted to compare the ZMF approach to fluoroscopy for the implantation of CIEDs. Therefore, this meta‐analysis was aimed to evaluate the feasibility and safety of the two approaches in navigating CIEDs implantation in adults.

## 2 MATERIALS AND METHODS

### 2.1 Study design

This systematic review and meta‐analysis were conducted based on the Preferred Reporting Items for Systematic Reviews and Meta‐Analyses (PRISMA) guidelines. This meta‐analysis has also been registered to PROSPERO (CRD42023414051).

### 2.2 Literature search

A literature search was conducted at three online databases (PubMed, ScienceDirect, and Web of Science) in March 2023. We used “zero fluoroscopy,” “cardiac pacemaker,” “cardiac pacing,” “cardiac implantable electronic device,” “cardiac resynchronization therapy,” “implantable cardioverter defibrillator,” and their synonyms as keywords during literature search, along with Boolean operators (AND, OR, and NOT). The reference lists of the studies were also screened to identify any additional appropriate article.

### 2.3 Eligibility criteria

All observational or experimental studies comparing the ZMF approach to conventional fluoroscopy for adult CIEDs implantation were included. Meanwhile, reviews, case reports/series, single‐arm, or animal studies were excluded. Articles published in non‐English languages were also excluded. The eligibility assessment was carried out by two independent authors (KL and CM). Disagreements between authors were resolved by the third author (RJ). Following articles with incomplete data, the author (KL or CM) contacted the corresponding author via email. If the corresponding author did not answer or decline the request within 14 days, the respective article was excluded.

### 2.4 Data extraction and collection

Two independent authors (KL and CM) extracted the following data for each article: authors‐publication year, location, patient(s) demographics, ZMF method, type of CIEDs, clinical indication, follow‐up duration, complications reported, success rate, procedural time, fluoroscopy time, and sum complication rate. The data presented in median and IQR were converted to mean and standard deviation according to the previous study.^6^ The data were collected into a dedicated spreadsheet.

### 2.5 Methodological quality assessment

Methodological quality assessment for the included articles was performed by two independent authors (KL and CM). Newcastle‐Ottawa Scale (NOS) was utilized for non‐randomized studies, otherwise modified Jadad scale was applied. The NOS is composed of three domains (Selection, Comparability, and Outcome), assessing the studies with a maximum score of 9. A score of 7–9 is considered good, 5–6 is fair, otherwise is poor studies. Meanwhile, the modified Jadad scale is composed of 8 yes/no questions. A score above 4 is considered a good‐quality study.

### 2.6 Outcome

The primary outcomes of this meta‐analysis were the success rate for the ZMF approach compared to the conventional fluoroscopy. Meanwhile, the secondary outcomes included procedural time, fluoroscopy time, radiation dose, and sum complications rate for the two.

### 2.7 Statistical analysis

Dichotomous variables (success rate and complication rate) were presented as odds ratio (OR), while continuous variables (procedural time and fluoroscopy time) were presented as standardized mean difference (SMD). An SMD of 0.2 represents a small effect, 0.5 represents a medium effect, and 0.8 or larger represents large effect.^7^ The heterogeneity was evaluated (subased on *I* ^2^ result. High heterogeneity is defined as *I* ^2^ above 50%, indicating the application of a random Mantel–Haenszel effect. A sensitivity analysis was carried‐out using the leave‐one‐out method to identify the etiology of heterogeneity. All statistical analysis was performed using RevMan 5.4. The result is considered significant if the *p* value scores less than .05, except for heterogeneity test (*p* < .10).

## 3 RESULTS

### 3.1 Included studies and baseline characteristics

**FIGURE 1:**
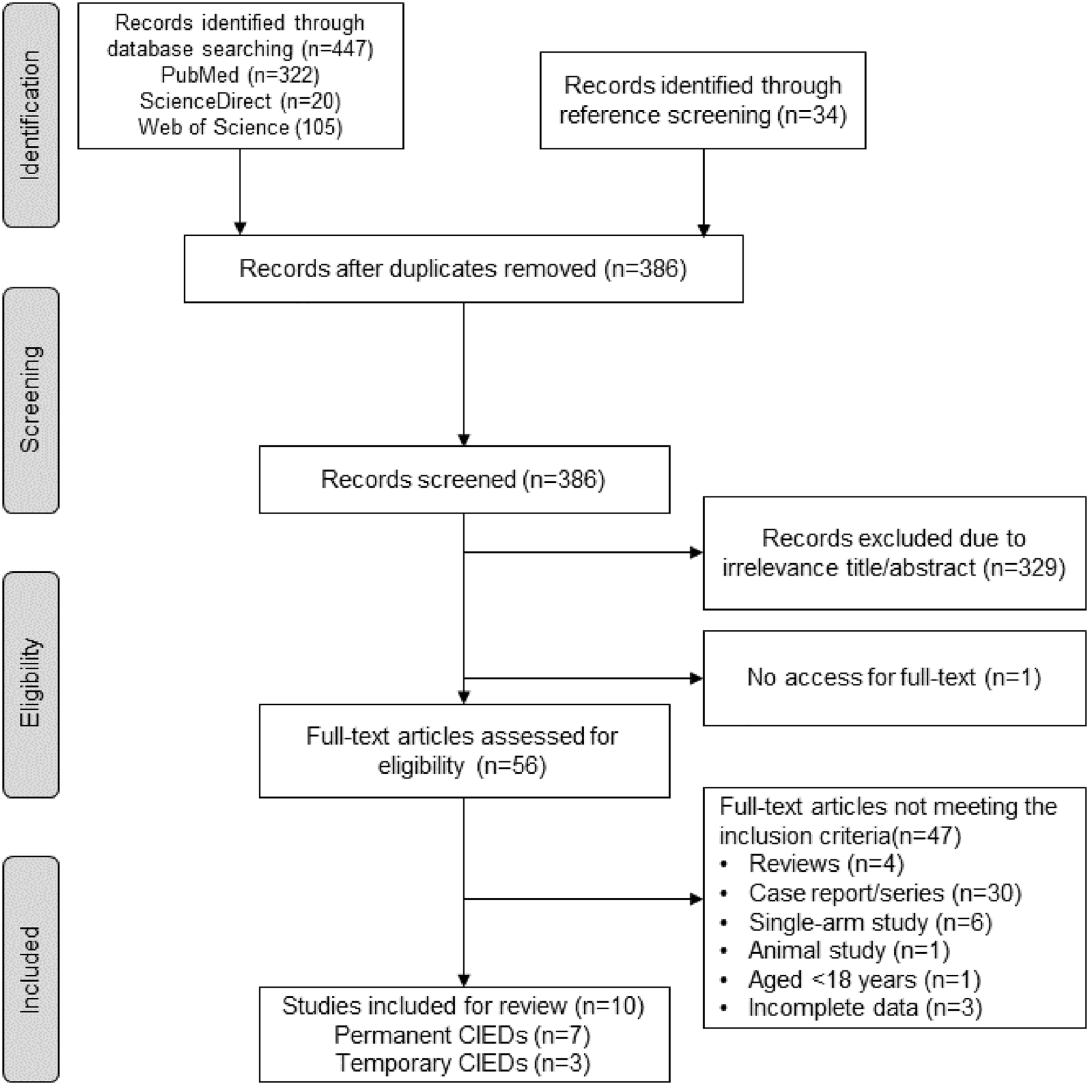
PRISMA flowchart.

The initial search resulted a total of 481 articles. After removing duplicates, 386 articles were screened, resulting into 56 relevant studies which were further assessed for eligibility. Finally, 7 articles with permanent CIEDs and 3 articles with temporary CIEDs were included for analysis (Figure 1).

Most participants were male and aged above 70 years old. Three‐dimensional electroanatomic mapping approach (3D‐EAM) and transthoracic echocardiography (TTE) were used in all permanent and temporary CIEDs implantation accordingly. Most studies included sinus node dysfunction (SND) and atrioventricular node block (AVB) as the clinical indications for CIEDs implantation (Table 1). Based on NOS criteria, all observational studies were considered good. However, the study by Ruiz‐Granell et al. (2008) did not provide a clear comparability analysis of its confounding factors (Table 2). One study by Hua et al. (2021), on the other hand, was evaluated using the modified Jadad scale and was considered good (6 of 8 points).

**TABLE 1:** Baseline characteristics of included studies.

Author Location (year)	Study design	Sample size (*n*)	Male (%)	Age (years)	ZMF methods	CIEDs	Clinical indication	Follow‐up duration	Complication reported
Permanent CIEDs									
Scarà et al. Italy (2022)^8^	MC Cohort	46 (ZMF 22/F 24)	63 (ZMF 68/F 58)	ZMF 79 ± 10 F 74 ± 8	KODEX‐EPD	HBP‐PPM HBP‐CRT	SND, AVB, CRT	1 and 6‐months	None
Hua et al. China (2021)^9^	RCT	20 (10/10)	65 (70/60)	55 ± 15.3 58 ± 16.2	KODEX‐EPD	HBP‐PPM	SND, AVB	3 months	Increased threshold (no intervention)
Richter et al. Germany (2020)^10^	SC Cohort	58 (29/29)	71 (69/72)	73 ± 13 71 ± 13	EnSite Precision	HBP‐PPM ICD	SND, AVB, CRT	1 month	Lead dislodgement, increased threshold
Sharma et al. USA (2019)^11^	MC Cohort	30 (10/20)	53 (60/50)	70 ± 14 72 ± 11	CARTO 3, EnSite Velocity	HBP‐PPM	SND, AVB	1 month	Increased threshold
Colella et al. Italy (2016)^12^	SC Cohort	61 (26/35)	72 (69/74)	72 ± 11 69 ± 7	EnSite Velocity	CRT‐P CRT‐D	HF‐requiring CRT	1, 6, and 36‐months	Lead dislodgement, infection, and death (at 36‐months follow‐up)
Del Greco et al. Italy (2017)^13^	MC Cohort	375 (125/250)	81 (80/82)	74 ± 4.5 75 ± 6.7	EnSite NavX	CRT‐P CRT‐D	Patients requiring CRT according to ESC 2013	Unspecified	CS dissection, lead dislodgement requiring repositioning, inappropriate shocks, pocket hematoma, pocket infection, acute kidney failure
Ruiz‐Granell et al. Spain (2008)^14^	Retro Cohort	30 (15/15)	n/a (40/n/a)	72 ± 13 n/a	EnSite NavX	sVDD‐PPM	AVB	3 months	Lead dislodgement
Temporary CIEDs									
Pinneri et al. Italy (2013)^15^	SC Cohort	106 (53/53)	52 (57/46)	77 ± 12 77 ± 9	Transthoracic Echocardiography	TPM	AVB, SND, TdP	24 h	PM malfunction
El Nasasra et al. Israel (2018)^16^	Retro Cohort	66 (17/49)	52 (71/45)	73 ± 13.2 75 ± 13.8	Transthoracic Echocardiography	TPM	AVB, SND, TdP, Asystole	Unspecified	Lead dislodgement, hematoma, Infection
Ferri et al. Italy (2016)^17^	SC Cohort	203 (113/90)	55 (55/54)	77 ± 6 77 ± 12	Transthoracic Echocardiography	TPM	AVB, SND, VT, Asystole	Unspecified	Infection, sepsis, hematoma. cardiac perforation, pneumothorax, sustained ventricular arrythmia, PM malfunction

Abbreviations: AVB, atrioventricular node block; CIEDs, cardiac implantable electronic devices; CS, coronary sinus; CRT, cardiac resynchronization therapy; CRT‐P/D, CRT‐pacemaker/defibrillator; F, fluoroscopy; HBP, His‐bundle pacing; HF, heart failure; ICD, implantable cardiac defibrillator; MC, multicenter; PPM, permanent pacemaker; RCT, randomized controlled trial; SC, single‐center; SND, sinoatrial node dysfunction; sVDD, single‐lead VDD; TdP, Torsades de Pointes; TPM, temporary pacemaker; VT, ventricular tachycardia; ZMF, zero/minimal fluoroscopy.

**TABLE 2:** Newcastle‐Ottawa Scale assessment.

Study	Selection	Comparability	Outcome	Result
Scarà et al. (2022)^8^	****	**	***	Good
Richter et al. (2020)^10^	****	**	***	Good
Sharma et al. (2019)^11^	****	**	***	Good
Colella et al. (2016)^12^	****	**	***	Good
Del Greco et al. (2017)^13^	****	**	***	Good
Ruiz‐Granell et al. (2008)^14^	****	n/a	***	Good
Pinneri et al. (2013)^15^	****	**	***	Good
El Nasasra et al. Israel (2018)^16^	****	**	***	Good
Ferri et al. Italy (2016)^17^	****	**	***	Good

### 3.2 Success rate

**FIGURE 2:**
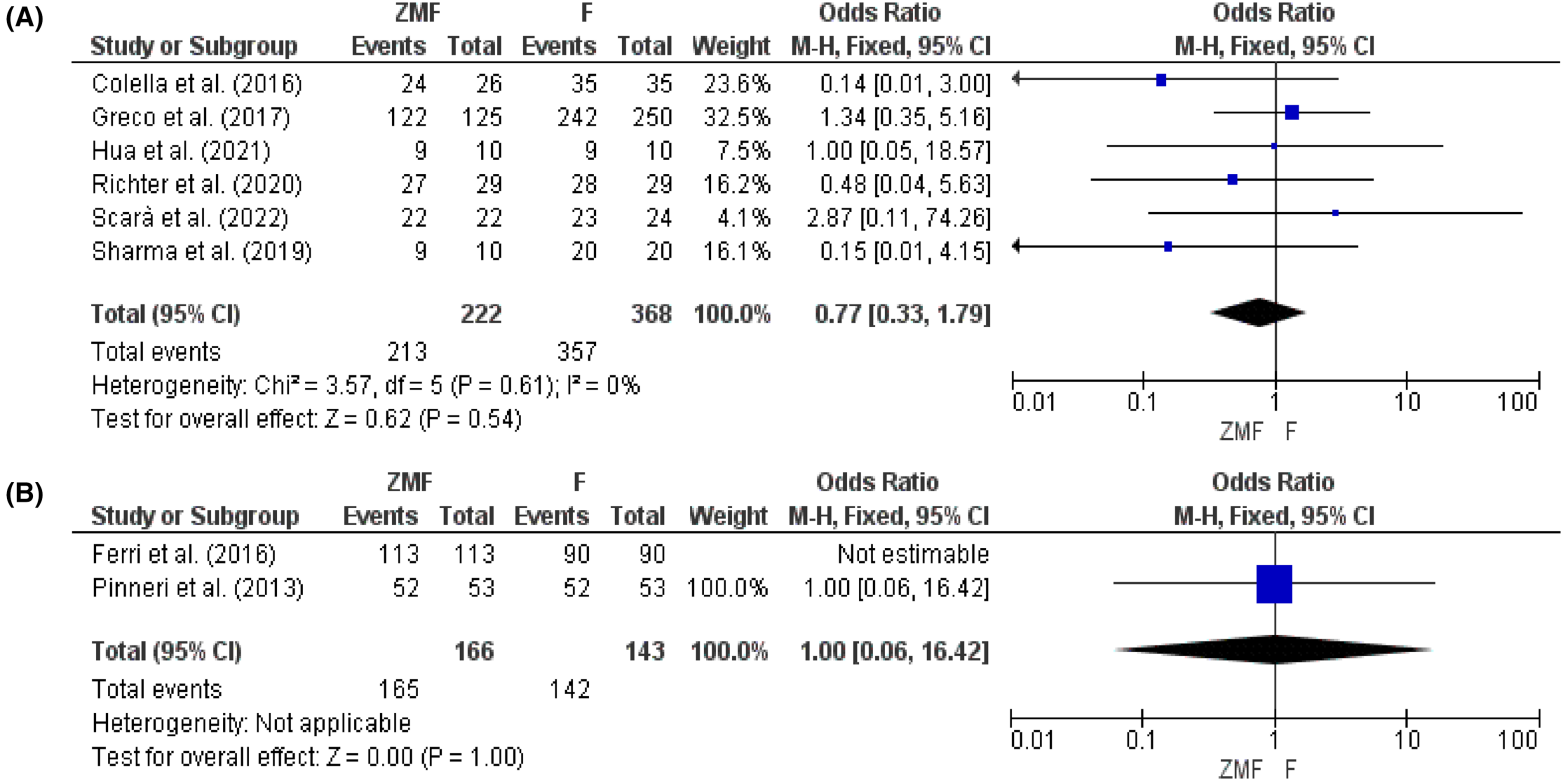
Success rate for (A) permanent and (B) temporary CIEDs.

Six studies reported the success rate of the ZMF approach for permanent CIEDs. The success rate of the ZMF approach was similar to the fluoroscopy method (OR: 0.77, 95% CI: 0.33–4.15, *p* = .54). The success rate for temporary CIEDs implantation using TTE was also comparable to the conventional approach, which ranged around 98%–100% (Figure 2).

### 3.3 Procedural time, fluoroscopy time, and radiation dose

**FIGURE 3:**
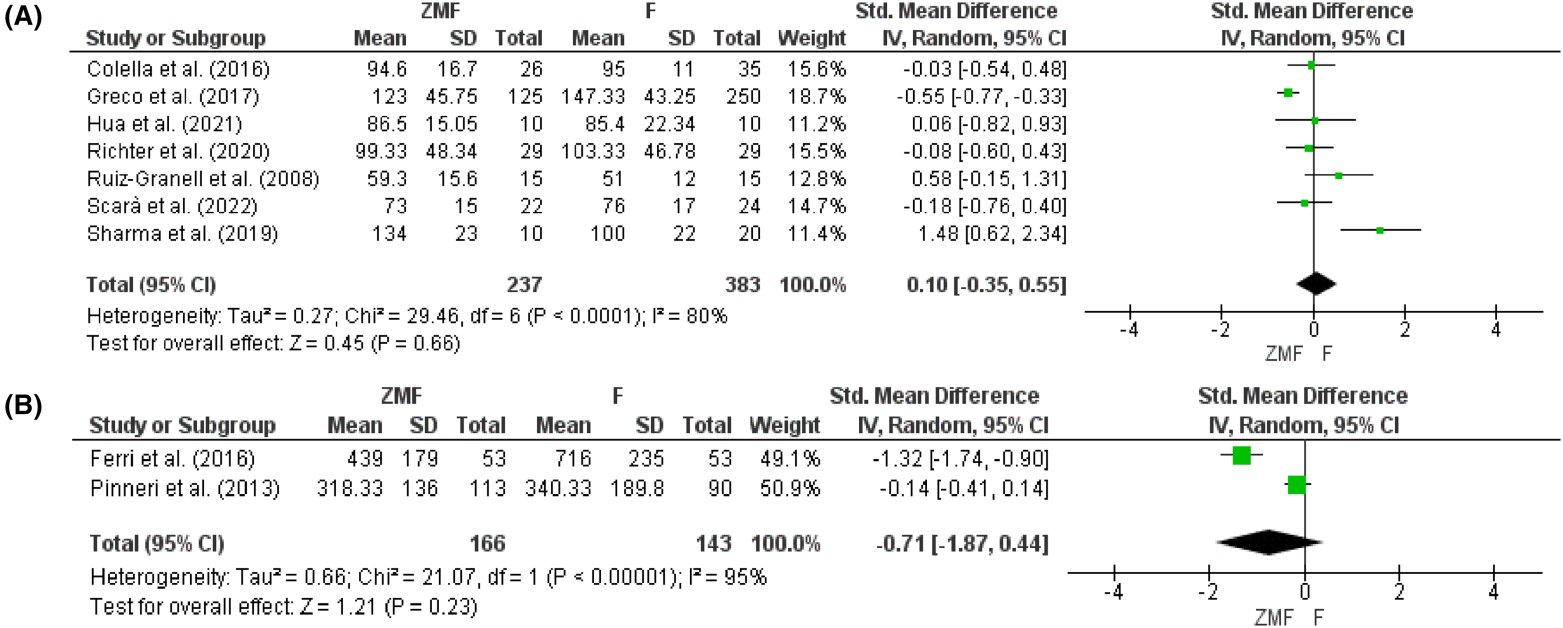
Procedural time for (A) permanent and (B) temporary CIEDs.

The procedural time (PT) for both permanent and temporary CIEDs implantation using the ZMF approach was similar compared to the fluoroscopy approach (SMD: 0.10, 95% CI: −0.35 to 0.55, *p* = .66 and SMD: −0.71, 95% CI: −1.87 to 0.44, *p* = .23, respectively) (Figure 3). The heterogeneity for permanent PT analysis was high (*I* ^2^ = 80%), so sensitivity analysis was carried out. After removing the study by Del Greco et al. (2017) or Sharma et al. (2019), heterogeneity was reduced to 62%, but the result remained insignificant (SMD: 0.24, 95% CI: −0.19 to 0.67, *p* = .28 or SMD: −0.12, 95% CI: −0.46 to 0.22, *p* = .50).

**FIGURE 4:**
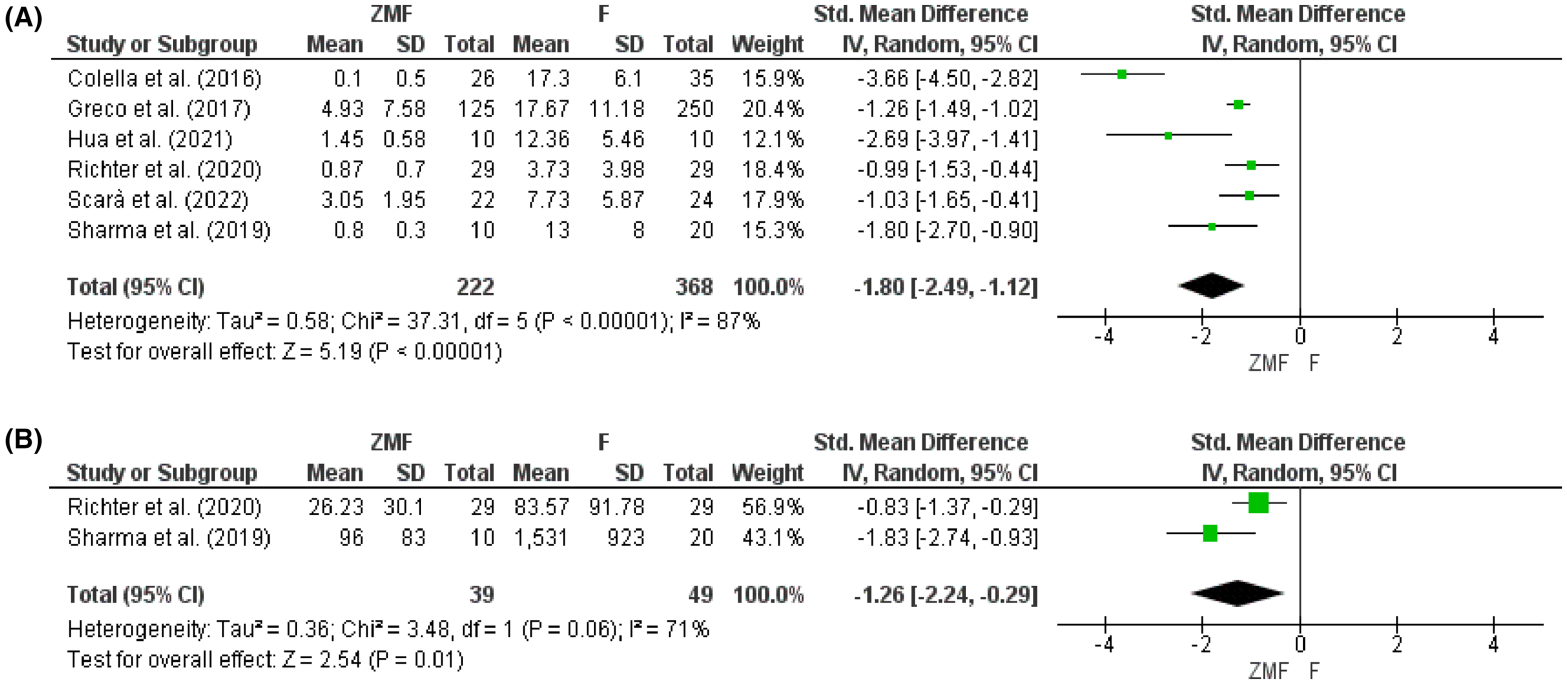
(A) Fluoroscopy time and (B) radiation dose for permanent CIEDs.

Contrary to PT, the fluoroscopy time (FT) and the radiation dose (RD) were shorter and lower in the ZMF approach (Figure 4). A significant reduction was observed for both FT and RD during permanent CIEDs implantation (SMD: −1.80, 95% CI: −2.49 to −1.12, *p* < .001 and SMD: −1.26, 95% CI: −2.24 to −0.29, *p* = .01). The result remained after sensitivity analysis by removing the study by Colella et al. (2016) with the heterogeneity of 48% (SMD: −1.32, 95% CI: −1.68 to −0.95, *p* < .001).

### 3.4 Complication rate

**FIGURE 5:**
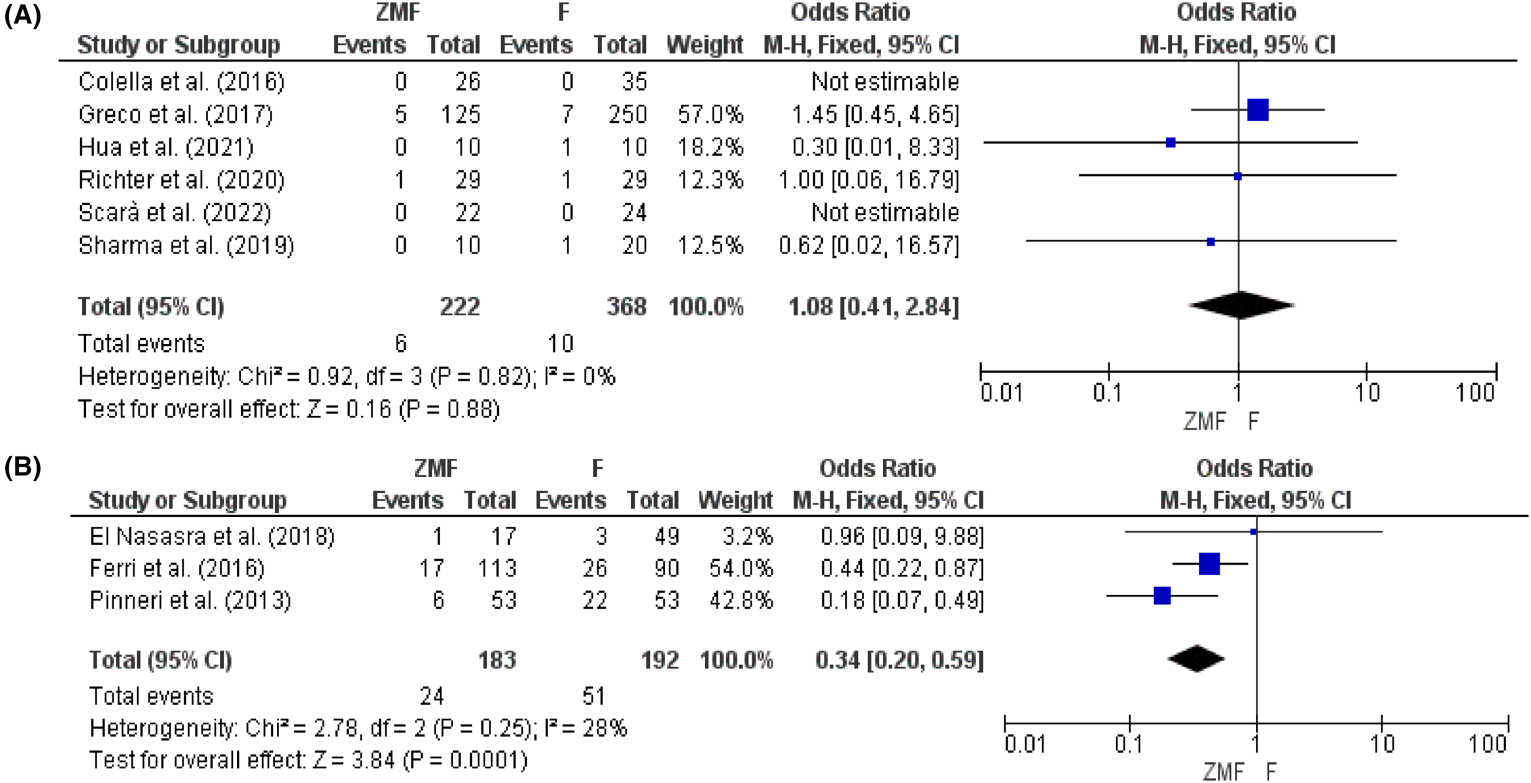
Sum complication rate for (A) permanent and (B) temporary CIEDs.

The complication rates for both ZMF and fluoroscopy approaches were relatively low. The overall complication of permanent CIEDs implantation using ZMF was not different from the fluoroscopy approach (OR: 1.08, 95% CI: 0.41–2.84, *p* = .88). The TTE guidance for CIEDs implantation was also found to be safer compared to the traditional fluoroscopy method (OR: 0.34, 95% CI: 0.20–0.59, *p* = .0001) (Figure 5).

## 4 DISCUSSION

Fluoroscopy is one of the most commonly used imaging modalities for numerous cardiac intervention procedures, including catheter ablation and CIEDs implantation. Despite its crucial role, fluoroscopy holds the risk of radiation exposure.^4^ Many approaches have been carried out to reduce radiation exposure, one of them using no or less fluoroscopy.

Many published articles reported the success of the ZMF approach for numerous cardiac procedures, such as catheter ablations and correction of congenital heart diseases. Arrhythmia termination using the ZMF approach (3D‐EAMS) was not inferior compared to conventional fluoroscopy.^5^, ^18^, ^19^ Meanwhile, transcatheter correction under echocardiography guidance could also be done to treat atrial septal defect and patent ductus arteriosus.^20^, ^21^ Interestingly, the ZMF approach had also been performed for CIEDs implantation.

To the best of our knowledge, this is the first meta‐analysis comparing the ZMF approach to conventional fluoroscopy for CIEDs implantation. The 3D‐EAMS was utilized for permanent CIEDs, while TTE was employed for temporary CIEDs implantation. Our results showed that not only the implantation under 3D‐EAMS guidance was comparable to conventional fluoroscopy in terms of success rate, PT, and complication rate but it also significantly reduced FT and RD. Lower complication rate was also observed in temporary CIEDs implantation using TTE approach.

3D‐EAMS reconstructs the geometry of cardiac chambers based on magnetic or impedance measurements. The 3D‐EAMS also provides precise position and movement of catheters along with the gradient of electrical activity in cardiac tissue.^22^ Currently, there are two most‐used 3D‐EAMS in the market: CARTO and EnSite.^23^ CARTO was the first 3D‐EAMS introduced by Biosense Webster (Johnson and Johnson). CARTO uses a magnetic field to reconstruct 3D images. However, the CARTO system is limited to only a few types of catheters.^24^ Meanwhile, EnSite (NavX and Velocity) reconstructs 3D images based on tissue impedance and is suitable with more catheter choices.^25^, ^26^ KODEX‐EPD, on the other hand, is the currently most novel 3D‐EAMS that utilizes dielectric characteristics to reconstruct 3D images.^27^

Despite our findings, we identified two barriers the 3D‐EAMS approach for CIEDs implantation. The first problem is the cost. The 3D‐EAMS method is 6% more expensive due to the additional use of decapolar catheters with magnetic sensors.^13^, ^28^ Currently, there was no comparison data regarding medical expenses of 3D‐EAMS and traditional fluoroscopy for CIEDs implantation. Castrejón et al. (2013) noted 3D‐EAMS cost more than traditional fluoroscopy due to the single use of 3D‐EAMS wear‐out components.^29^ Economic analysis study on SVT catheter ablation suggested that 3D‐EAMS potentially increases the cost by $207‐3.454 (2014‐USD).^30^, ^31^ The 2019 Asia Pacific Heart Rhythm Society (APHRS) consensus also agreed that the 3D‐EAMS method is very beneficial for the patients, yet is limited to its availability and financial issues. Therefore, 3D‐EAMS should be offered to selective population, respectively.^22^ Another barrier includes operator experience since 3D‐EAMS is a relatively new method, especially for CIEDs implantation. One study mentioned that 3D‐EAMS procedures took longer than the conventional approach, even for experienced operators. However, as the operators experienced more cases, the overall procedure time was found to be reduced.^32^ Another study also showed that 26 cases were sufficient to reduce procedural time by 2.3 times (136–59 min).^12^ This outcome suggests that the ZMF approach has the potential to be the guiding option for CIED insertion that is as effective and efficient as the fluoroscopy approach with a potential operator‐dependence barrier.

### 4.1 Benefits for clinical practice

ZMF guiding for CIEDs implantation provides benefits for both patients and medical staffs. The magnitude of fluoroscopy radiation depends on the clinical indications, comorbidities, and device type.^33^, ^34^ Patients with long, multiple, and complicated procedures had higher risks of radiation injury, especially skin injuries (20 min or more of high contrast fluoroscopy or 60 min of low fluoroscopy).^4^ As important as the patients, medical staffs, namely interventional cardiologists, electrophysiologists, and cardiac catheterization laboratory technicians also had an increased risk of radiation‐associated injuries, such as skin lesions, orthopedic problems, cataracts, and hypercholesterolemia.^35^, ^36^ The ZMF approach also allows interventional cardiac procedures for susceptible populations, such as pregnant women, pediatrics, and those with chronic kidney disease or allergic reaction to contrast.^18^

### 4.2 Limitations of the study

This review has several limitations. First, this review did not include all identified studies exploring the ZMF approach for CIEDs implantation. Although three studies had fulfilled our eligibility criteria, the reported data were not complete enough to be analyzed. We had contacted the corresponding author, but there was no response.^32^, ^37^, ^38^ However, the results of these studies were linear to our results. Second, this review only included 995 participants (620 and 375 participants for permanent and temporary CIEDs, respectively), which was relatively small to draw sufficient conclusions. Seventy percent of the studies had a sample size of less than 70. Third, fluoroscopic measurements, such as radiation dosage and contrast volume, were underreported in most studies. Lastly, it is important to recognize that the generalizability of these findings might be constrained due to the limited focus of the included studies, one of them being the lack of data regarding pregnancy status. This variable is crucial, as pregnant patients benefited the most from this novel approach. The only available studies reporting successful CIEDs implantation using 3D‐EAM among pregnant patients were in case report designs.^39^, ^40^, ^41^, ^42^, ^43^, ^44^, ^45^ Therefore, further studies should aim to address the limitations of our meta‐analysis and systematic review by including a more diverse range of study populations to confirm the feasibility and safety of the ZMF approach for CIEDs implantation, especially among pregnant patients.

## 5 CONCLUSIONS

Zero to minimal fluoroscopy approach had a similar success rate, procedural time, and sum complication rate for permanent CIEDs implantation with significant reductions of fluoroscopy time and radiation exposure. A more minimal complication rate was observed in temporary CIEDs implantation using transthoracic echocardiography compared to fluoroscopy with comparable procedural time.

## FUNDING INFORMATION

No funding was received for the production of this manuscript.

## CONFLICT OF INTEREST STATEMENT

Authors declare no conflict of interests for this article.

## META‐ANALYSIS REGISTRATION

PROSPERO (CRD42023414051).

## Data Availability

All data underlying the results are available as part of the article and no additional source data are required.

## References

[1] Willy K , Ellermann C , Reinke F , Rath B , Wolfes J , Eckardt L , et al. The impact of cardiac devices on patients' quality of life—a systematic review and meta‐analysis. J Cardiovasc Dev Dis. 2022;9(8):257. 36005421 10.3390/jcdd9080257 PMC9409697

[2] Hussein AA , Wilkoff BL . Cardiac implantable electronic device therapy in heart failure. Circ Res. 2019;124(11):1584–1597. 31120815 10.1161/CIRCRESAHA.118.313571

[3] Ali M , Banavalikar B , Kanjwal K , Ghadei MK , Kottayan A , Padmanabhan D , et al. Effect of fluoroscopy frame rate on radiation exposure and in‐hospital outcomes in cardiovascular implantable electronic device implantation procedures. Radiat Med Prot. 2021;2(4):176–180.

[4] Picano E , Piccaluga E , Padovani R , Antonio Traino C , Grazia Andreassi M , Guagliumi G . Risks related to fluoroscopy radiation associated with electrophysiology procedures. J Atr Fibrillation. 2014;7(2):1044. 27957094 10.4022/jafib.1044 PMC5135251

[5] Chiang LLW , Li C , Hong KL , Hui WS , Beh SY , Gong M , et al. The use of minimal fluoroscopy for cardiac electrophysiology procedures: a meta‐analysis and review of the literature. Clin Cardiol. 2021;44(6):814–823. 33998690 10.1002/clc.23609 PMC8207968

[6] Wan X , Wang W , Liu J , Tong T . Estimating the sample mean and standard deviation from the sample size, median, range and/or interquartile range. BMC Med Res Methodol. 2014;14(1):1–13. 25524443 10.1186/1471-2288-14-135 PMC4383202

[7] Takeshima N , Sozu T , Tajika A , Ogawa Y , Hayasaka Y , Furukawa TA . Which is more generalizable, powerful and interpretable in meta‐analyses, mean difference or standardized mean difference? BMC Med Res Methodol. 2014;14(1):30. 24559167 10.1186/1471-2288-14-30 PMC3936842

[8] Scarà A , Golia P , Grieco D , Borrelli A , de Ruvo E , Bressi E , et al. Low fluoroscopy permanent His bundle pacing using a new electroanatomic mapping system (KODEX EPD). A multicenter experience. J Arrhythm. 2022;39(1):18–26. 36733331 10.1002/joa3.12803 PMC9885313

[9] Hua W , Liu X , Gu M , Niu H , Chen X , Tang M , et al. Novel wide‐band dielectric imaging system guided lead deployment for his bundle pacing: a feasibility study. Front Cardiovasc Med. 2021;8:1–11. 10.3389/fcvm.2021.712051 PMC8446512 34540916

[10] Richter S , Ebert M , Bertagnolli L , Gebauer R , Lucas J , Scheller D , et al. Impact of electroanatomical mapping‐guided lead implantation on procedural outcome of His bundle pacing. Europace. 2021;23(3):409–420. 33253376 10.1093/europace/euaa292

[11] Sharma PS , Huang HD , Trohman RG , Naperkowski A , Ellenbogen KA , Vijayaraman P . Low fluoroscopy permanent His bundle pacing using electroanatomic mapping: a feasibility study. Circ Arrhythm Electrophysiol. 2019;12(2):1–9. 10.1161/CIRCEP.118.006967 30704289

[12] Colella A , Giaccardi M , Colella T , Modesti PA . Zero x‐ray cardiac resynchronization therapy device implantation guided by a nonfluoroscopic mapping system: a pilot study. Hear Rhythm. 2016;13(7):1481–1488. 10.1016/j.hrthm.2016.03.021 26976037

[13] Del Greco M , Maines M , Marini M , Colella A , Zecchin M , Vitali‐Serdoz L , et al. Three‐dimensional electroanatomic mapping system‐enhanced cardiac resynchronization therapy device implantation: results from a multicenter registry. J Cardiovasc Electrophysiol. 2017;28(1):85–93. 27862594 10.1111/jce.13120

[14] Ruiz‐Granell R , Ferrero A , Morell‐Cabedo S , Martinez‐Brotons A , Bertomeu V , Llacer A , et al. Implantation of single‐lead atrioventricular permanent pacemakers guided by electroanatomic navigation without the use of fluoroscopy. Europace. 2008;10(9):1048–1051. 18523029 10.1093/europace/eun139

[15] Pinneri F , Frea S , Najd K , Panella S , Franco E , Conti V , et al. Echocardiography‐guided versus fluoroscopy‐guided temporary pacing in the emergency setting: an observational study. J Cardiovasc Med. 2013;14(3):242–246. 10.2459/JCM.0b013e32834eecbf 22240748

[16] El Nasasra A , Alnsasra H , Zahger D , Lerman TT , Kobal S , Cafri C , et al. Feasibility and safety of exclusive echocardiography‐guided intravenous temporary pacemaker implantation. J Echocardiogr. 2019;17(3):157–161. 30426465 10.1007/s12574-018-0406-4

[17] Ferri LA , Farina A , Lenatti L , Ruffa F , Tiberti GL , Piatti L , et al. Emergent transvenous cardiac pacing using ultrasound guidance: a prospective study versus the standard fluoroscopy‐guided procedure. Eur Hear J Acute Cardiovasc Care. 2016;5(2):125–129. 10.1177/2048872615572598 25673783

[18] Debreceni D , Janosi K , Vamos M , Komocsi A , Simor T , Kupo P . Zero and minimal fluoroscopic approaches during ablation of supraventricular tachycardias: a systematic review and meta‐analysis. Front Cardiovasc Med. 2022;9:856145. 35479287 10.3389/fcvm.2022.856145 PMC9037593

[19] Yang L , Sun G , Chen X , Chen G , Yang S , Guo P , et al. Meta‐analysis of zero or near‐zero fluoroscopy use during ablation of cardiac arrhythmias. Am J Cardiol. 2016;118(10):1511–1518. 27639689 10.1016/j.amjcard.2016.08.014

[20] Ackermann S , Quandt D , Hagenbuch N , Niesse O , Christmann M , Knirsch W , et al. Transcatheter atrial septal defect closure in children with and without fluoroscopy: a comparison. J Interv Cardiol. 2019;2019:6598637. 31772540 10.1155/2019/6598637 PMC6739773

[21] Siagian SN , Prakoso R , Putra BE , Kurniawati Y , Lelya O , Sembiring AA , et al. Echocardiography‐guided percutaneous patent ductus arteriosus closure: 1‐year single center experience in Indonesia. Front Cardiovasc Med. 2022;9:885140. 35677684 10.3389/fcvm.2022.885140 PMC9167953

[22] Kim YH , Chen SA , Ernst S , Guzman CE , Han S , Kalarus Z , et al. 2019 APHRS expert consensus statement on three‐dimensional mapping systems for tachycardia developed in collaboration with HRS, EHRA, and LAHRS. J Arrhythm. 2020;36(2):215–270. 32256872 10.1002/joa3.12308 PMC7132207

[23] Qiu J , Wang Y , Chen G , Zhao C , Wang DW . Progress in zero‐fluoroscopy implantation of cardiac electronic device. Pacing Clin Electrophysiol. 2020;43(6):609–617. 32348595 10.1111/pace.13930

[24] Maury P , Monteil B , Marty L , Duparc A , Mondoly P , Rollin A . Three‐dimensional mapping in the electrophysiological laboratory. Arch Cardiovasc Dis. 2018;111(6):456–464. 29887403 10.1016/j.acvd.2018.03.013

[25] Eitel C , Hindricks G , Dagres N , Sommer P , Piorkowski C . EnSite velocity cardiac mapping system: a new platform for 3D mapping of cardiac arrhythmias. Expert Rev Med Devices. 2010;7(2):185–192. 20214424 10.1586/erd.10.1

[26] Romero J , Lupercio F , Goodman‐Meza D , Ruiz JC , Briceno DF , Fisher JD , et al. Electroanatomic mapping systems (CARTO/EnSite NavX) vs. conventional mapping for ablation procedures in a training program. J Interv Card Electrophysiol. 2016;45(1):71–80. 26560500 10.1007/s10840-015-0073-6

[27] Ding L , Huang X , Dai C , Zhang H , Weng S , Yu F , et al. Safety and effectiveness of a novel dielectric mapping system: one‐year, two chinese centers experiences. BMC Cardiovasc Disord. 2022;22(1):352. 35922759 10.1186/s12872-022-02790-8 PMC9351078

[28] Hofer D , Steffel J , Duru F , Graup V , Sasse T , Saguner A , et al. Feasibility, efficiency, and safety of zero‐fluoroscopy catheter interventions for right‐sided cardiac arrhythmias using only electroanatomic mapping. Cardiology. 2022;147(5–6):547–556. 35977529 10.1159/000526564 PMC9808658

[29] Castrejón‐Castrejón S , Pérez‐Silva A , González‐Villegas E , Al‐Razzo O , Silvestre J , Doiny D , et al. Implantation of cardioverter defibrillators with minimal fluoroscopy using a three‐dimensional navigation system: a feasibility study. Europace. 2013;15(12):1763–1770. 23696625 10.1093/europace/eut127

[30] Hindricks G , Willems S , Kautzner J , de Chillou C , Wiedemann M , Schepel S , et al. Effect of electroanatomically guided versus conventional catheter ablation of typical atrial flutter on the fluoroscopy time and resource use: a prospective randomized multicenter study. J Cardiovasc Electrophysiol. 2009;20(7):734–740. 19298568 10.1111/j.1540-8167.2009.01439.x

[31] Marini M , Ravanelli D , Martin M , Del Greco M , Guarracini F , Quintarelli S , et al. An economic analysis of the systematic use of mapping systems during catheter ablation procedures: single center experience. Biomed Res Int. 2019;2019:2427015. 31531347 10.1155/2019/2427015 PMC6720348

[32] Patel H , Hiner E , Naqvi A , Wrobel J , Machado C . The safety and efficacy of electroanatomical mapping (EAM)‐guided device implantation. Pacing Clin Electrophysiol. 2019;42:897–903. 31106434 10.1111/pace.13724

[33] Alkhorayef M , Sulieman A , Babikir E , Daar E , Alnaaimi M , Alduaij M , et al. Patient exposure during fluoroscopy‐guided pacemaker implantation procedures. Appl Radiat Isot. 2018;138:14–17. 28830729 10.1016/j.apradiso.2017.08.010

[34] Attanasio P , Lacour P , Ernert A , Pieske B , Haverkamp W , Blaschke F , et al. Cardiac device implantations in obese patients: success rates and complications. Clin Cardiol. 2017;40(4):230–234. 28333397 10.1002/clc.22650 PMC6490533

[35] Andreassi MG , Piccaluga E , Guagliumi G , Del Greco M , Gaita F , Picano E . Occupational health risks in cardiac catheterization laboratory workers. Circ Cardiovasc Interv. 2016;9(4):e003273. 27072525 10.1161/CIRCINTERVENTIONS.115.003273

[36] Stahl CM , Meisinger QC , Andre MP , Kinney TB , Newton IG . Radiation risk to the fluoroscopy operator and staff. Am J Roentgenol. 2016;207(4):737–744. 28829623 10.2214/AJR.16.16555

[37] Mina A , Warnecke N . Near zero fluoroscopic implantation of BIV ICD using electro‐anatomical mapping. Pacing Clin Electrophysiol. 2013;36(11):1409–1416. 23902517 10.1111/pace.12221

[38] Guo P , Qiu J , Wang Y , Chen G , Proietti R , Fadhle AS , et al. Zero‐fluoroscopy permanent pacemaker implantation using Ensite NavX system: clinical viability or fanciful technique? Pacing Clin Electrophysiol. 2018;41(2):122–127. 29222861 10.1111/pace.13248

[39] Velasco A , Velasco VM , Rosas F , Cevik C , Morillo CA . Utility of the NavX® electroanatomic mapping system for permanent pacemaker implantation in a pregnant patient with chagas disease. Indian Pacing Electrophysiol J. 2013;13(1):34–37. 23329872 10.1016/s0972-6292(16)30586-1 PMC3539398

[40] Tuzcu V , Kilinc OU . Implantable cardioverter defibrillator implantation without using fluoroscopy in a pregnant patient. Pacing Clin Electrophysiol. 2012;35(9):1–2. 21955026 10.1111/j.1540-8159.2011.03221.x

[41] Pedrinazzi C , Gazzaniga P , Durin O , Tovena D , Inama G . Implantation of a permanent pacemaker in a pregnant woman under the guidance of electrophysiologic signals and transthoracic echocardiography. J Cardiovasc Med. 2008;9(11):1169–1172. 10.2459/JCM.0b013e3283100edc 18852597

[42] Payne J , Lo M , Paydak H , Maskoun W . Near‐zero fluoroscopy implantation of dual‐chamber pacemaker in pregnancy using electroanatomic mapping. Hear Case Rep. 2017;3(4):205–209. 10.1016/j.hrcr.2016.12.008 PMC5419812 28491803

[43] Kuhne M , Schaer B , Reichlin T , Sticherling C , Osswald S . X‐ray‐free implantation of a permanent pacemaker during pregnancy using a 3D electro‐anatomic mapping system. Eur Heart J. 2015;36(41):2789–272790. 10.1093/eurheartj/ehv234 26040799

[44] Chua KCM , Lim ETS , Chong DTT , Tan BY , Ho KL , Ching CK . Implantation of a dual‐chamber permanent pacemaker in a pregnant patient guided by intracardiac echocardiography and electroanatomic mapping. Hear Case Rep. 2017;3(11):542–545. 10.1016/j.hrcr.2017.09.003 PMC5688237 29204351

[45] Hartz J , Clark BC , Ito S , Sherwin ED , Berul CI . Transvenous nonfluoroscopic pacemaker implantation during pregnancy guided by 3‐dimensional electroanatomic mapping. Hear Case Rep. 2017;3(10):490–492. 10.1016/j.hrcr.2017.07.020 PMC5643851 29062705

